# Psoriasis patients demonstrate HLA-Cw*06:02 allele dosage-dependent T cell proliferation when treated with hair follicle-derived keratin 17 protein

**DOI:** 10.1038/s41598-018-24491-z

**Published:** 2018-04-17

**Authors:** Milyausha Yunusbaeva, Ruslan Valiev, Fanil Bilalov, Zilya Sultanova, Leyla Sharipova, Bayazit Yunusbayev

**Affiliations:** 1 Institute of Biochemistry and Genetics Ufa Federal Research Centre of the Russian Academy of Sciences, 450054 Ufa, Bashkortostan Russia; 2 Bashkir State Pedagogical University, 450000 Ufa, Bashkortostan Russia; 3 Bashkir State University, 450076 Ufa, Bashkortostan Russia; 4 Department of Laboratory Diagnostic, Bashkir State Medical University, 450003 Bashkortostan, Russia; 5 Republic Dermatovenerologic Dispensary, 450010 Bashkortostan, Russia; 6 Evolutionary Biology group, Estonian Biocentre, Institute of Genomics, University of Tartu, 51010 Tartu, Estonia

## Abstract

It is broadly accepted that psoriasis is an immune-mediated disease with a heritable component, but it is not clear what causes inflammation in the skin. Previous research suggests that fragments of the keratin 17 (K17) protein, which are constitutively expressed in hair follicles, could act as autoantigens. In this study, we synthesized the K17 protein from mRNA derived from hair follicles and tested whether it elicited T cell responses depending on the patient genotype at the major susceptibility locus HLA-Cw*06:02. We treated peripheral blood-derived cells with the K17 protein and its short fragments to assess the T cell proliferation response using flow cytometry. Our analyses show a significantly stronger increase in cell proliferation among patients but not in healthy controls. We then examined whether the variation in T cell proliferation correlated with the patient HLA-Cw*06:02 risk genotype. Considering the affected status and patient genotype as two independent predictors, we fitted a linear model and showed that the HLA-Cw*06:02 allele dosage strongly predicted the T cell response. Our study findings suggest that the K17 protein likely acts as an autoantigen in psoriasis and that patients’ risk genotype is strongly correlated with the magnitude of the response to this putative autoantigen.

## Introduction

Psoriasis is an immune-mediated chronic skin disease with a complex aetiology involving both genetic risk factors and environmental triggers. Skin lesions in psoriasis are infiltrated by inflammatory cells, but a marked increase in proliferation and turnover of keratinocytes distinguishes psoriasis from other inflammatory skin diseases. As the major site of inflammation, the skin is in continuous communication with the systemic immune, neural and endocrine systems and is capable of recognizing, discriminating and integrating various signals within a highly heterogeneous environment^1^. Although various environmental factors have been reported to influence psoriasis^2^, only infections with beta-haemolytic streptococci have been convincingly associated with both the initiation and exacerbation of psoriasis^3–5^. There is evidence that a streptococcal throat infection accompanies a considerable fraction of psoriasis cases, ranging from 56% to 60% according to some reports^6,7^. Moreover, blood samples of psoriasis patients often contain antibodies to beta haemolytic streptococci and the pathogen itself^8^. Therefore, the putative mechanism of inflammation could be similar to those observed in acute rheumatic fever and rheumatic heart disease. In this disease, streptococcal superantigen (GAS) circulating in the infected organism disrupts host immune tolerance and increases infiltration of GAS activated immune cells into the target tissue^9^. An autoimmune response then develops because GAS-activated immune cells erroneously respond to self-proteins through a process called molecular mimicry^10^. In the case of psoriasis, host antigens must be specific to the skin and joints, and keratin 17 (K17) is the most studied candidate to date^11–14^. This protein shares short peptide motifs with the M protein, a virulence factor of Beta-haemolytic streptococcal infection^15^. The expression level of K17 protein is normally low but increases in response to injury during tissue repair and cell division^16,17^. Interestingly, in this regard, the protein is constitutively expressed in hair follicles and nail beds - two sites that often first develop psoriasis lesions^14^.

Streptococcal infections are known to affect a large proportion of the human population, and yet, only 2–5% of the general population develops psoriasis. One possible explanation for this discrepancy might be a variable genetic predisposition. Genetically susceptible individuals may have an altered immune response leading to the persistence of a streptococcal infection in their body. At the same time, they may also have a higher predisposition to mount a stronger immune response once their immune tolerance is disrupted, for example, by stress.

In this study, we genotyped the major genetic susceptibility HLA-Cw*06:02 locus^18^ in psoriasis patients and healthy controls and studied their cell-based immune response against the putative autoantigen K17. Specifically, we evaluated whether hair follicle-derived keratin 17 can elicit an immune response in psoriasis patients and whether their genotype at the HLA-Cw*06:02 locus can predict the strength of the response to this putative autoantigen.

## Results

### Reverse Transcription PCR, cloning and expression of recombinant proteins

We obtained full-length K17 cDNA from hair follicles of psoriatic patients as well as two small fragments of the *KRT17* gene, which we refer to as S1 and S4. These small proteins were previously shown to stimulate interferon-gamma production in psoriatic T cells^12^. Altogether, three recombinant plasmids were constructed, which we refer to as pGEX4T1-K17, pGEX4T1-S1 and pGEX4T1-S4. We verified successful insertion of target fragments using restriction analysis (Supplementary Fig. S1) and DNA sequencing, which showed no alterations in our targeted gene sequence.

Verified pGEX4T1-K17, pGEX4T1-S1 or pGEX4T1-S4 plasmids were successfully transformed into *E*. *coli* BL21 cells, and bacterial cells were induced to express recombinant proteins using IPTG (isopropyl-beta-d-thiogalactopyranoside). The resulting glutathione-S-transferase (GST)-fusion proteins were purified with glutathione sepharose 4B affinity columns. SDS-PAGE (sodium dodecyl sulfate polyacrylamide gel electrophoresis) analysis confirmed that purified proteins were synthesised as a soluble fraction (Supplementary Fig. S2). After cleaving GST-tagged tails, we performed immunoblotting and identified the protein molecular sizes to be approximately 47 kDa, 6 kDa and 8 kDa for K17, S1 and S4 (Supplementary Fig. S3).

### Proliferation assay

**Figure 1:**
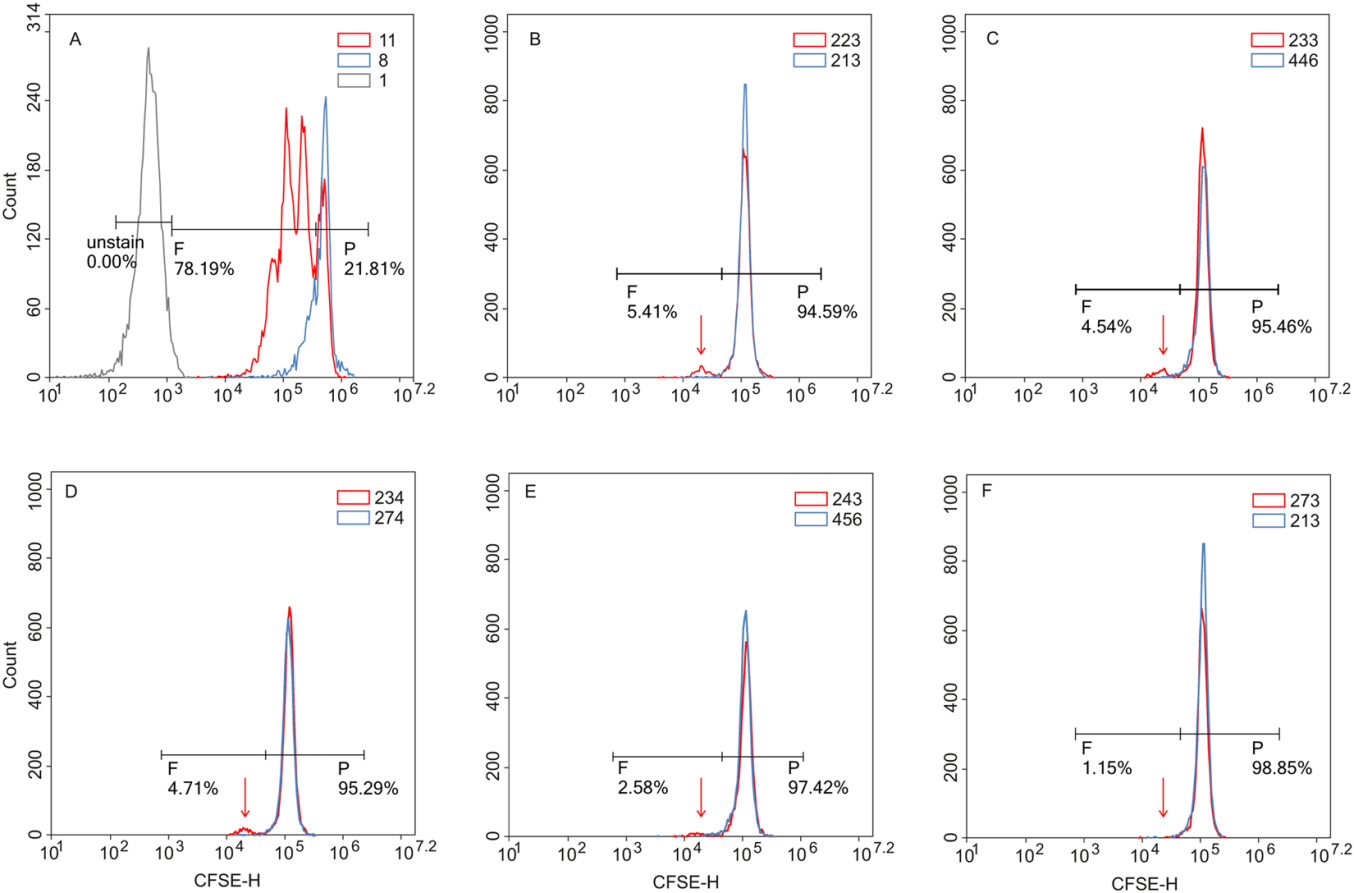
*In vitro* T cell proliferation. Mononuclear cells from psoriatic patients (red curve) and healthy controls (blue curve) were labelled with 1 µM CFSE prior to culturing and incubated for 5 days alone, with 1 µg/ml PHA (**A**) or in the presence of recombinant proteins K17 (**B**), S1 (**C**), S4 (**D**), PS1 (**E**) and PK (**F**) at concentration of 10 µg/ml. After 5 days, cells were labelled with a PE-conjugated anti-CD3 antibody and 7-AAD prior to flow cytometry analysis. Gated CD3+ lymphocytes are shown on the CFSE fluorescence histograms to demonstrate the decrease in fluorescence intensity during divisions. The higher peak for both patients and healthy controls represents a larger population of non-dividing parental cells (P). The signal of interest is the smaller peak that corresponds to dividing cells (shown using arrow under **F**), and such cells are only observed in patents when treated with K17 (**B**), S1 (**C**), or S4 (**D**) proteins. Figures represent the percentage of CFSE low CD3+ cells (proliferating population).

**Figure 2:**
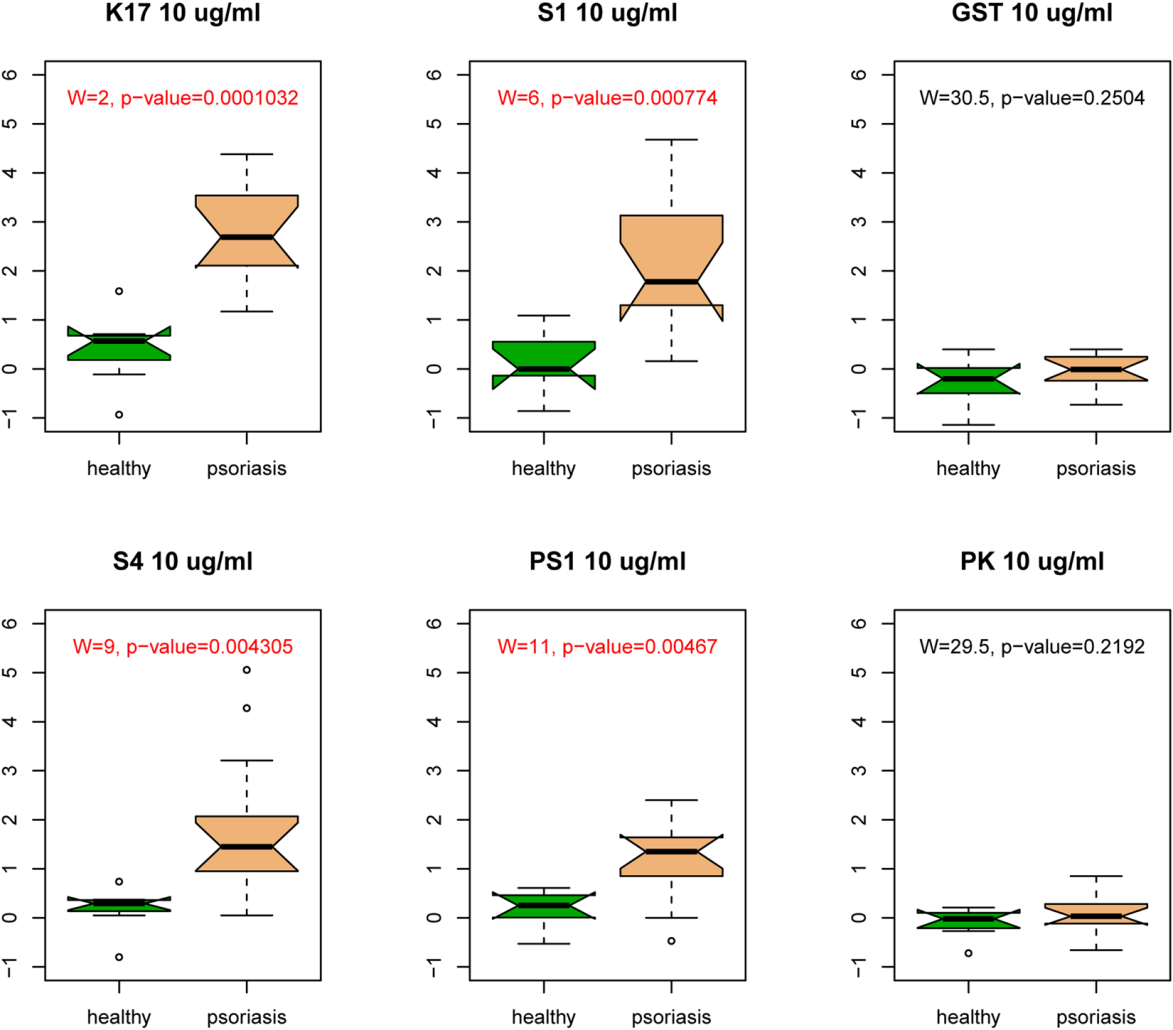
Comparative analysis of the T cell proliferation response between psoriasis patients and healthy donors. Boxplots show the T cell proliferation response when treated with K17, the S1 fragment of K17, GST, the S4 fragment of K17, or two synthetic peptides, PS1 and PK. Statistically significant differences between patients and controls are shown in red. The Mann-Whitney test, also known as the Wilcoxon rank sum test, is given as W together with the corresponding p-value.

To assess whether recombinant K17 could elicit a T cell response, we treated peripheral blood mononuclear cells (PBMCs) derived from patients and healthy controls with this protein for five days. The same experiment was carried out using the S1 and S4 K17 fragments. In addition to the recombinant proteins, we also tested two synthesized peptides, VRALEEANTELEVKI and its scrambled version АTAGVAAVVKRKEEN, that represent K17 fragments, which we referred to as PS1 and PK, respectively. Phytohemagglutinin (PHA) was used as a positive control and the glutathione-S-transferase (GST) protein as a negative control. All cell stimulation experiments were carried out at protein concentrations of 10 μg/ml and 100 μg/ml, and the proliferation response was measured using a CFSE (carboxyfluorescein) flow cytometry assay (Fig. 1, Table 1). Table 1 shows the average proportion of dividing cells in patients and controls. These flow cytometry based raw estimates were then corrected by subtracting blank measurements, and differences between patients and controls were assessed using the Mann-Whitney test (also known as the Wilcoxon rank sum test). At both concentrations (10 μg/ml and 100 μg/ml), the full-length K17 protein demonstrated a markedly stronger proliferative effect (W = 2, p = 0.0001 and W = 1, p = 0.000005, respectively) on T cells derived from patients compared to those from control individuals (Fig. 2, Supplementary Fig. S5). Because experiments at both concentrations gave qualitatively similar outcomes (Fig. 2, Supplementary Fig. S5), from here onward, we present only the results obtained at concentrations of 10 μg/ml. Stimulation with the S1 and S4 K17 fragments also induced stronger T cell proliferation (W = 6, p = 0.0007 and W = 9, p = 0.004, respectively) in psoriasis patients than in healthy controls (Fig. 2). As expected, we observed no difference in proliferative activity between patients and healthy controls (W = 30.5, p = 0.25) when cells were treated with the GST protein. Finally, we also confirmed that the PS1 peptide induced a stronger response in patients cells compared to healthy control cells (W = 11, p = 0.004), whereas the scrambled peptide PK did not show a statistically significant effect (W = 29.5, p = 0.219).

**Table 1:** Average proportion of proliferating cells in patients and controls as measured by flow cytometry.

Protein, µg/ml	K17, 10	K17, 100	S1, 10	S1, 100	S4, 10	S4, 100	PS1, 10	PS1,100	PK, 10	PK, 100	GST, 10	GST, 100
**Controls (n = 15)**	1.0 ± 0.5	1.3 ± 0.5	0.8 ± 0.5	0.9 ± 0.2	0.8 ± 0.2	0.9 ± 0.1	0.8 ± 0.2	0.9 ± 0.1	0.5 ± 0.1	0.9 ± 0.3	0.3 ± 0.4	0.4 ± 0.4
**Patients (n = 29)**	3.2 ± 0.9	3.9 ± 0.9	2.7 ± 1.5	3.2 ± 1.6	2.2 ± 1.5	2.8 ± 1.5	1.6 ± 0.8	2.1 ± 1.1	0.5 ± 0.4	0.6 ± 0.4	0.4 ± 0.4	0.6 ± 0.5

Average percentage of dividing cells without blank subtraction and standard deviation (mean ± s.d.).

### HLA-Cw*06:02 allele dose-dependent T cell proliferation

The HLA-Cw*06:02 allele is a major predisposing genetic factor for psoriasis in various world populations^19–22^, and we first evaluated its frequency in our study cohort. Consistent with its important role in psoriasis susceptibility, we observed a significantly higher incidence of the HLA-Cw*06:02 allele among patients (53.85%) compared to healthy controls (14.29%) (p = 0.01986, Fisher’s exact test).

To study T cell proliferation as a function of an individual’s genotype, we first added disease status (affected or healthy) as a predictor into our linear regression model. Consistent with our Mann-Whitney test, there is a statistically significant relationship between disease status and the T cell proliferation response (Beta-coefficient = 2.4, p-value = 0.000075), and disease status explained 57% of the variability in response (Adjusted R-squared = 0.57, p-value = 0.000074). We then extended our linear model by adding the HLA-Cw*06:02 allele dosage (0, 1, and 2) as an additional explanatory variable. The fitting of this two-variable model showed that the HLA-Cw*06:02 risk allele dosage had a strong and independent effect on the proliferation response (Beta-coefficient = 1.16, p-value = 0.00109) in addition to the disease status effect (Beta-coefficient = 1.48, p-value = 0.00272). Moreover, our results suggest that knowledge of the HLA-Cw*06:02 genotype substantially increases our confidence in predicting (Adjusted R-squared = 0.76, p-value = 0.00002068) the T-cell proliferation response compared to when only the disease status is known (Adjusted R-squared = 0.57).

## Discussion

In this study, we show that full length keratin 17 elicits a strong T cell proliferation response in psoriasis patients and that proliferation intensity correlates with the patient genotype at the HLA-Cw*06:02 locus. Our results support the idea that endogenous peptides that have similarity to streptococcal M-proteins^11,12,14,15^ can act as autoantigens in psoriasis. It should be noted that endogenous proteins other than keratin 17, such as maspin, ezrin, PRDX2, hsp27, and potentially, keratin 6 have been previously shown to act as autoantigens in a streptococcal-induced autoimmune response^23^. A recent study by Lande R. *et al*.^24^, for example, showed that the antimicrobial peptide LL37 likely acts as a T-cell autoantigen in psoriasis. They also tested keratin 17 and keratin 6, but unlike our findings, only a small proportion of their patients (26% and 8.7% of patients, respectively) exhibited a response to these self-proteins. One possible explanation for this discrepancy might be the different proportion of HLA-Cw*06:02 carriers among their patients. A recent study identified a nonapeptide autoantigen from skin-specific melanocytes in an HLA-Cw*06:02 positive psoriasis patient^25^. They further confirmed this autoantigen in 20 out of 42 patients for which genotypes at the HLA-C*06:02 locus were not reported. Thus, there is increasing evidence that multiple autoantigens in different skin-specific cells, rather than a single major peptide, trigger inflammation in psoriasis.

T cell-based experiments reported to date do not always evaluate the role of patient genotype at the HLA-C*06:02 locus. Meanwhile, our results in addition to those of other reports suggest that the allele dosage at this risk locus is positively correlated with the strength of the T cell response, which is an important immunological phenotype. The likely molecular mechanism underlying this immunological phenotype was proposed in a recent study by Wei and colleagues^26^. They examined the structure of the receptor-ligand complex formed by the HLA-C*06:02 allele product and pVR autoantigen peptide from psoriasis patients^26^. According to their results, the HLA-C*06:02 allele product, unlike other proteins encoded by the alternative *HLA-C* alleles, leads to a much stronger bond in the complex because of the two pockets with negative potential in the HLA-C*06:02 allele product and two corresponding basic residues at position 2 (P2) and position 7 (P7) within the pVR peptide. Importantly, these biochemical properties are shared with the peptides in our study that showed a T cell response and by other responsive autoantigens reported in Lande *et al*.^24^, while they are lacking in non-responsive peptides^26^. Thus, stronger receptor-ligand complexes and TCR signalling could explain the stronger T cell response with the increasing HLA-C*06:02 allele dosage observed in our study. This adverse effect of the HLA-C*06:02 allele has a likely evolutionary explanation since *HLA-C* alleles were under natural selection during human evolution^27^. Specifically, it is hypothesized that the immunological phenotype in psoriasis was advantageous against invasive streptococcal infections in the past^4,5^. This connection between individuals’ risk genotype and resistance to historically important infections is a promising area for further research.

## Materials and Methods

### Ethics statement

All subjects provided informed consent for this study, which was approved by the Ethics Committee of the Institute Biochemistry and Genetics Ufa Federal Research Centre of the Russian Academy of Sciences (IBG UFRC RAS). This project was performed in accordance with the approved guidelines from the Ethics Committee.

### Patients and control volunteers

Twenty-nine patients with moderate-to-severe chronic plaque psoriasis were selected from the Republican Dermatovenerologic Dispensary in Ufa (Republic of Bashkortostan, Russia). The inclusion criteria included a PASI index of 10–19.9, corresponding to the medium-severe course of the disease. The mean age of patients was 43 ± 15.7 years, including 51.7% males (15 people) and 48.3% females (13 people). Psoriatic patients were either untreated or had only received topical therapy during the last four weeks prior to sampling. Fifteen healthy volunteers without dermatological diseases, matched for age (mean age 41 ± 12.5 years) and sex (8 men (53.3%) and 7 women (46.7%)), were selected for all experiments. Ten millilitres of venous blood was obtained from patients with chronic plaque psoriasis and healthy volunteers.

### RNA extraction from hair follicles

The following protocol was approved by the Ethics Committee of the Institute Biochemistry and Genetics Ufa Federal Research Centre of the Russian Academy of Sciences. After receiving full informed consent, three to five hair follicles were extracted using sterile forceps from inflamed, psoriatic areas of patient scalps. The bottom portion of each hair (0.5 centimetre in length), containing the hair follicle, was trimmed off into a 2 ml microcentrifuge tube containing 1 ml of STE buffer (10 мМ Tris НСl (pH 7.5), 100 мM NaCl, 1 мМ EDTA Na, pH 8), 10% sodium dodecyl sulfate (SDS), proteinase K (2 mg/ml) and RNase inhibitor (RiboLock RNase inhibitor, 20 U/ml)). Samples were incubated at 37 °C for 1 hour. Total RNA was extracted using Trizol Reagent according to manufacturer’s instructions (Invitrogen Life Technologies, Carlsbad, CA, USA), and the quality of isolated RNA was evaluated using 1% agarose gel electrophoresis.

### RT-PCR and construction of expression vectors

cDNA was reverse transcribed from total RNA, and three PCR fragments (K17, S1, and S4) were amplified using AmpliTaq Gold DNA polymerase (Life Technologies) using the following primers: K17 (F 5′ATGGATCCCTCTCCAGCCCTTCTCCT-3′ (*Bam*HI site underlined), R 5′ATGAATTCTCAGGCAAGGAAGCATG-3′ (*Eco*RI site underlined); primers for S1 region (F 5′ATGGATCCATGGGTGGTGGCTATGGCAGCA-3′, R 5′ CGGAATTCTCAGTACCAGTCACGGATCTTCA-3′), and S4 region (F 5′ATGGATCCATGACCATGCAGGCCTTGGAGA-3′, R 5′AGGAATTCTCACGTCTTCACATCCAGCAGGA-3′)^12^. Sequence analysis was performed using the Vector NTI software package. PCR was performed at 95 °C for 5 min (one cycle), 94 °C for 45 s, 60 °C for 45 s, 72 °C for 1 min (30 cycles), 72 °C for 5 min and 4 °C hold for K17 and at 95 °C for 5 min (one cycle), 94 °C for 30 s, 57 °C for 30 s, 72 °C for 35 s (28 cycles) 72 °C for 3 min and 4 °C hold for both S1 and S4 epitope regions. PCR product lengths were 1485, 189 and 232 bp for K17, S1, and S4, respectively. Purified PCR products were cloned into the pGEX4T1 vector using *Bam*HI and *Eco*RI (Fermentas) restriction enzymes. Successful recombination was confirmed by restriction enzyme digestion and DNA sequencing. The plasmids containing the inserts were named pGEX4T1-K17, pGEX4T1-S1 and pGEX4T1-S4.

### Expression, purification, and identification of the K17 and predicted epitope regions

Fusion proteins were expressed using *E*. *coli* BL21 (GE Healthcare). A single colony was inoculated into 5 ml of LB medium containing ampicillin (50 μg/ml) and incubated overnight at 37 °C in the orbital shaker. Next, the overnight culture was inoculated into fresh Lysogeny broth medium (LB) (1% volume/volume) containing a final concentration of 100 µg/ml ampicillin and incubated at 37 °C at 250 rpm until the culture reached an optical density at 600 nm of 0.6–0.8. Protein expression was induced by addition of 0.1 mМ IPTG followed by incubation for an additional 3 h at 37 °C for K17 and 2 hours at 28 °C for the S1 and S4 fragments. Pellets were resuspended in 30 ml of ice-cold STE buffer with phenylmethylsulfonyl fluoride (PMSF, 1 mM), dithiothreitol (DTT, 1 mM) and protease inhibitor cocktail. All steps were performed at 4 °C. Cells were subjected to sonication (25 pulses for every 30 seconds), and Triton X-100 was added to a final concentration of 2% as previously reported^12^. The obtained mixtures were centrifuged (12,000 g, 10 min) at 4 °C, and supernatants with extracted proteins were loaded to a pre-equilibrated glutathione sepharose 4B for 30 min following the manufacturer’s directions. The glutathione treated suspensions were transferred to pre-equilibrated columns, and GST-fusion proteins were rinsed as described previously^28^. GST-fusion proteins were eluted using elution buffer (50 mM Tris-HCl pH 8.0, 10 mM glutathione) and analysed using 12% SDS-PAGE. The pGEX4T1 plasmid lacking the insert was used as a control.

Immunoblotting was performed after transferring to polyvinylidene difluoride (PVDF) membranes according to the standard experimental protocol. The following antibodies were used for immunoblotting: a mouse anti-human keratin 17 monoclonal antibody (MCA1872, Bio-Rad) and anti-GST monoclonal antibody (HCA054A, Bio-Rad). A rabbit anti-mouse polyclonal antibody conjugated to horseradish peroxidase (STAR13B, Bio-Rad) was used for detection. GST-fused protein cleavage was performed with thrombin as recommended by the enzyme manufacturer (Amersham Biosciences). Residual enzyme activity was removed by affinity chromatography on HiTrap Benzamidine FF columns (GE Healthcare) according to the manufacturer’s instructions. The concentrations of recombinant proteins (K17, S1, S4, and GST) were calculated by the Bradford method. The final working concentration of each recombinant protein (10 μg/ml and 100 μg/ml in all the experiments) was established by investigating the cytotoxic effects. The cytotoxic test was carried out on peripheral blood mononuclear cells (PBMCs) of healthy volunteers using the 7-Aminoactinomycin D (7AAD) cell viability assay. Isolated PBMCs were incubated with the tested proteins for 24 h at varying concentrations ranging between 1 to 300 μg/ml. None of the tested protein concentrations affected the viability of PBMCs.

In addition to recombinant proteins, we used the synthesized peptide VRALEEANTELEVKI and its scrambled version АTAGVAAVVKRKEEN to represent K17 fragments. We refer to these peptides as PS1 and PK throughout the text. The PS1 peptide has been previously shown to stimulate proliferation and interferon-gamma production in psoriatic T cells^12^. Peptides were synthesized by Almabion Ltd (Russia). Lyophilized peptides were resuspended in PBS at a stock concentration of 1 mg/ml. The final concentration for each peptide was 10 μg/ml or 100 μg/ml in all experiments described.

### Cell isolation, T-cell stimulation and CFSE analysis

Human PBMCs were prepared from heparinized venous blood by Ficoll Paque (Sigma-Aldrich) density gradient centrifugation. Freshly isolated PBMCs were labelled with 1 µM CFSE (carboxyfluorescein) in phosphate-buffered saline (PBS) containing 0.1% BSA (Bovine Serum Albumin) (Sigma-Aldrich) for 10 min at room temperature in the dark and washed three times with cold PBS/0.1% BSA according to the manufacturer’s directions. Cells were resuspended in T-cell medium (RPMI 1640, 10% heat-inactivated foetal bovine serum (Sigma), 2 mM L-glutamine, 10 U/ml penicillin and 100 mg/ml streptomycin) and then incubated at a concentration of 1 × 10^5^ cells/well alone or in the presence of the tested proteins/peptides at 37 °C. All experiments were performed in triplicate. Phytohemagglutinin (PHA) was used as a positive control (Biolot, 1 μg/mL). Cells were incubated for five days and then stained with the antibodies CD3-PE (Cat. No. 12-0036-42) and CD45-PE-Cy5 (Cat. No. 15-0459-42) according to the manufacturer’s instructions (eBioscience). The 7-AAD viability solution (7-Aminoactinomycin D, Cat. No. 00-6993050, eBioscience) was used to remove dead cells from CFSE stained cells. The background level settings were determined based on the signals from isotype (Cat. No. 12-4714-41, 15-4714-41 eBioscience) control stained cells. Analysis of CFSE-labelled cells was performed by flow cytometry (NovoCyte™ 2000 Flow Cytometry System, ACEA). Lymphocytes were gated for analysis by a combination of a forward and side light scatter (FSC and SSC, respectively) and phenotypic T cell marker (CD3). For each sample analysed, at least 15,000 events were acquired in the gate. All downstream analysis steps were performed using NovoExpress and ModFit software (Verity Software House, Topsham, ME).

### HLA-Cw*06:02 genotyping

Genomic DNA was extracted from peripheral blood by phenol-chloroform extraction. The HLA-Cw*06:02 (*rs1050414*) polymorphism was analysed as described by R. T. Ahnini^29^. The 618-bp PCR product was cleaved into fragments of 348 and 270 bp in non-HLA-Cw*06:02 individuals, whereas the HLA-Cw*06:02 allele resulted in fragments of 348, 270, 196 and 74 bp in heterozygous individuals and 348, 196 and 74 bp in homozygous individuals (Supplementary Fig. S4).

### Statistical Analysis

To carry out statistical analyses for proliferation data, we corrected the percent of proliferating cells as measured by flow cytometry by subtracting blank measurements (the amount of spontaneously dividing cells in the same medium used). The Stats R package^30^ was used to carry out the Mann-Whitney test (also known as the Wilcoxon rank sum test) and to assess whether patients had a higher percentage of proliferating T cells compared to controls when treated with K17 protein; its S1 and S4 subregions; and the PS1, GST and KP proteins. The HLA-Cw*06:02 risk allele frequency and genotype frequency differences between patients and controls were tested using the Fishers’ exact test using the *fisher*.*test* R function. We evaluated a possible linear relationship between the HLA-Cw*06:02 risk allele dosage and the T cell proliferation response by fitting a linear model using the *lm* function in the R package^30^. We then fitted a linear model (with corresponding p-value) in which cell proliferation represented a quantitative response to disease status and risk allele dosage.

### Data Availability

All of the data analysed during this study are available upon request to the authors.

## Electronic supplementary material

Supplementary Dataset 1
