# Supplementary material for: Psoriasis patients demonstrate HLA-Cw*06:02 allele dosage-dependent T cell proliferation when treated with hair follicle-derived keratin 17 protein
Source: Sci Rep. 2018 Apr 17;8:6098. doi: 10.1038/s41598-018-24491-z (PMC5904118; doi:10.1038/s41598-018-24491-z)
Supplement: Supplementary file 1 — Supplementary Dataset 1 [file 41598_2018_24491_MOESM1_ESM.doc]

**Supplementary Information**

**Psoriasis patients demonstrate *HLA-Cw*06:02* allele dosage-dependent T cell proliferation when treated with hair follicle-derived keratin 17 protein**

Milyausha Yunusbaeva^1,2^, Ruslan Valiev^3^, Fanil Bilalov^4^, Zilya Sultanova^5^, Leyla Sharipova^5^, Bayazit Yunusbayev^1,6^ *.

^1^ Institute of Biochemistry and Genetics Ufa Federal Research Centre of the Russian Academy of Sciences, 450054, Ufa, Bashkortostan, Russia

^2^ Bashkir State Pedagogical University, 450000, Ufa, Bashkortostan, Russia

^3^ Bashkir State University, 450076, Ufa, Bashkortostan, Russia

^4^ Department of Laboratory Diagnostic, Bashkir State Medical University, 450003, Bashkortostan, Russia

^5^  Republic Dermatovenerologic Dispensary, 450010, Bashkortostan, Russia

^6^ Evolutionary Biology group, Estonian Biocentre, Institute of Genomics, University of Tartu 51010, Tartu, Estonia

*** Corresponding author**. Yunusbayev Bayazit

[bayazit@ebc.ee](mailto:bayazit@ebc.ee), [yunusbb@gmail.](mailto:yunusbb@inbox.ru)com

**Affiliations** Evolutionary Biology group, Estonian Biocentre, Institute of Genomics, University of Tartu, Tartu, Estonia; Institute of Biochemistry and Genetics Ufa Federal Research Centre of the Russian Academy of Sciences, 450054, Ufa, Bashkortostan, Russia


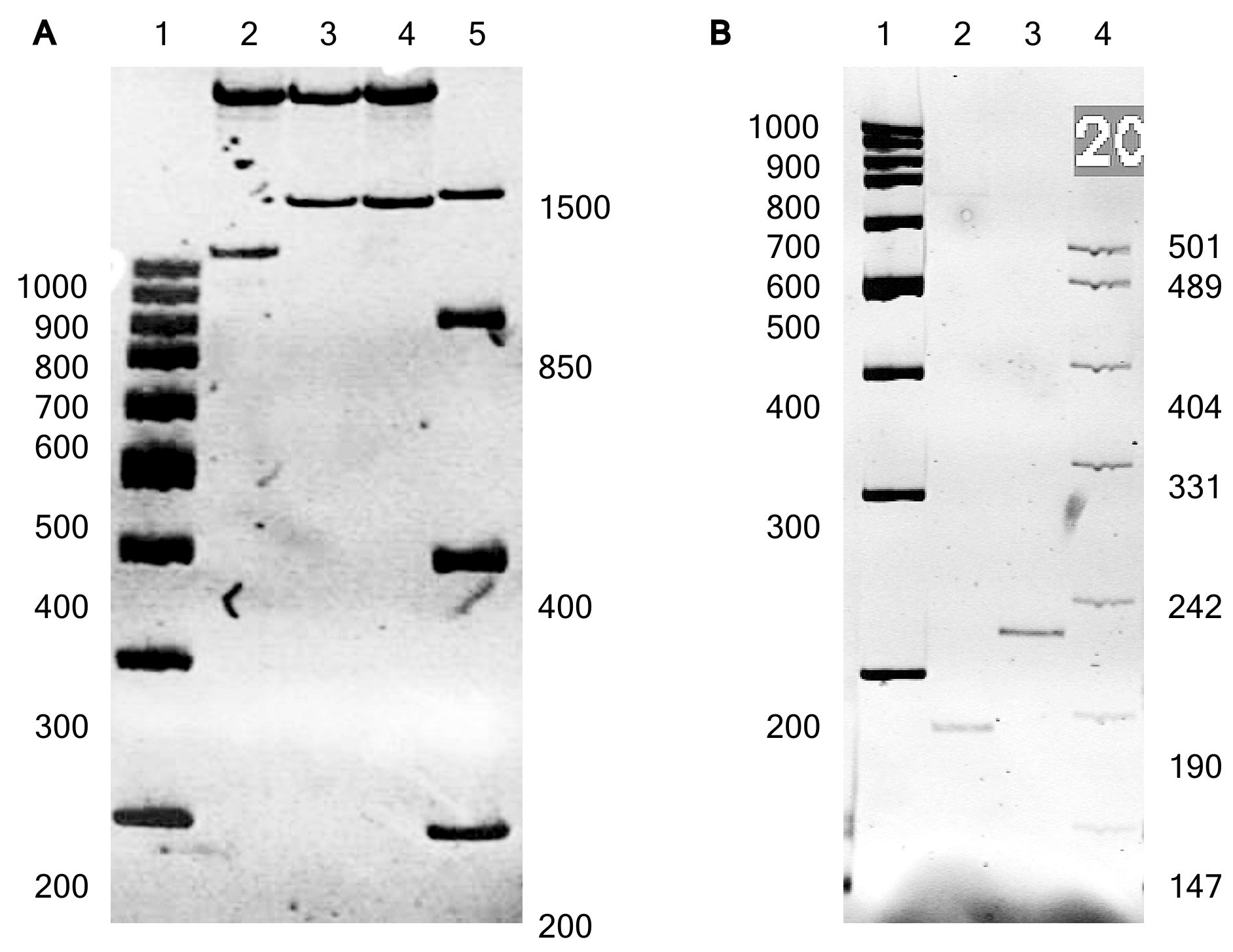


**Supplementary Figure S1.**

**A. Confirmation of the insertion of the K17 cDNA cloned in the pGEX4T1 vector at the *Bam*HI and *Eco*RI restriction sites.** Lane 1 is the GeneRuler 100 bp DNA Ladder (Fermentas), lanes 2-4 are clones that presumably contain an insert of K17 cDNA digested with *Bam*HI and *Eco*RI enzymes: Lane 2 is slightly shorter than expected insert (approximately 1000 bp), lanes 3 and 4 correspond to clones having 1485 bp long K17 insert, which is theoretically expected. Lane 5 is the FastRuler ™ DNA Ladder (Fermentas).

**B. Confirmation of the S1 and S4 insertions in the pGEX4T1 vector at the *Ba*mHI and *Eco*RI restriction sites.** Lane 1 is the GeneRuler 100 bp DNA Ladder (Fermentas), Lane 2 corresponds to clone having the S1 insert (189 bp), and lane 3 corresponds to clone having the S4 insert (232 bp). Lane 4 is the pUC19 DNA/*Msp*I (*Hpa*II) marker.


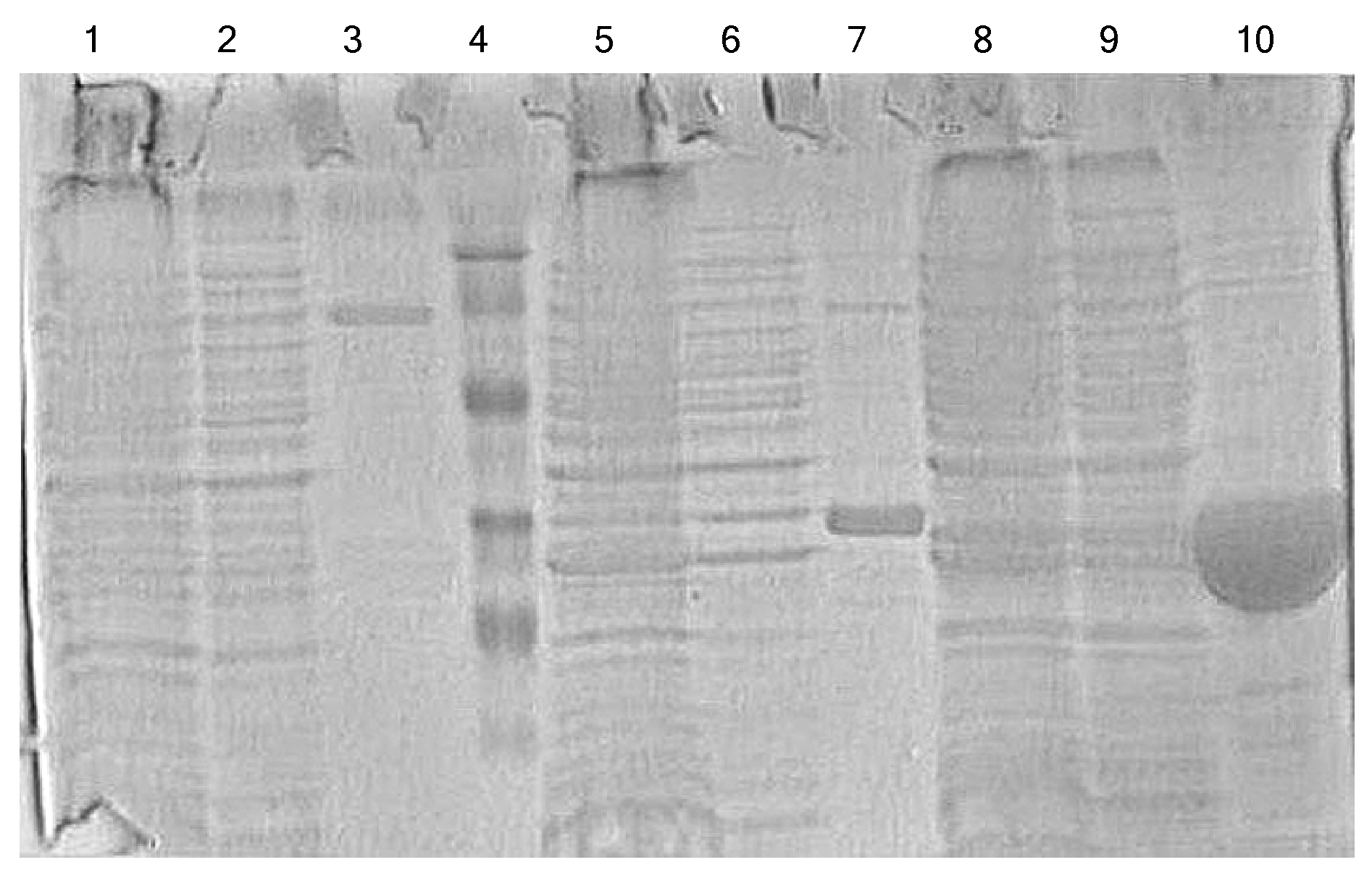


**Supplementary Figure S2. SDS-PAGE electrophoresis of purified recombinant protein.** Lane 1 – whole cell lysate of *E.coli* BL21 transformed with pGEX4T-K17, Lane 2 - flow through fraction after incubation with glutathione sepharose 4B, Lane 3 - elution fraction of proteins GST-K17 (73 kDa), Lane 4 - Prestained Protein Molecular Weight Market (SM0441, Fermentas), Lane 5 – whole cell lysate of *E.coli* BL21 transformed with pGEX4T-S1, Lane 6 - flow through fraction, Lane 7 - elution fraction of proteins GST-S1 (34 kDa), Lane 8 - whole cell lysate of *E.coli* BL21 transformed with pGEX4T-S4, Lane 9 - flow through fraction, Lane 10 - elution fraction of proteins GST-S4 (32 kDa).


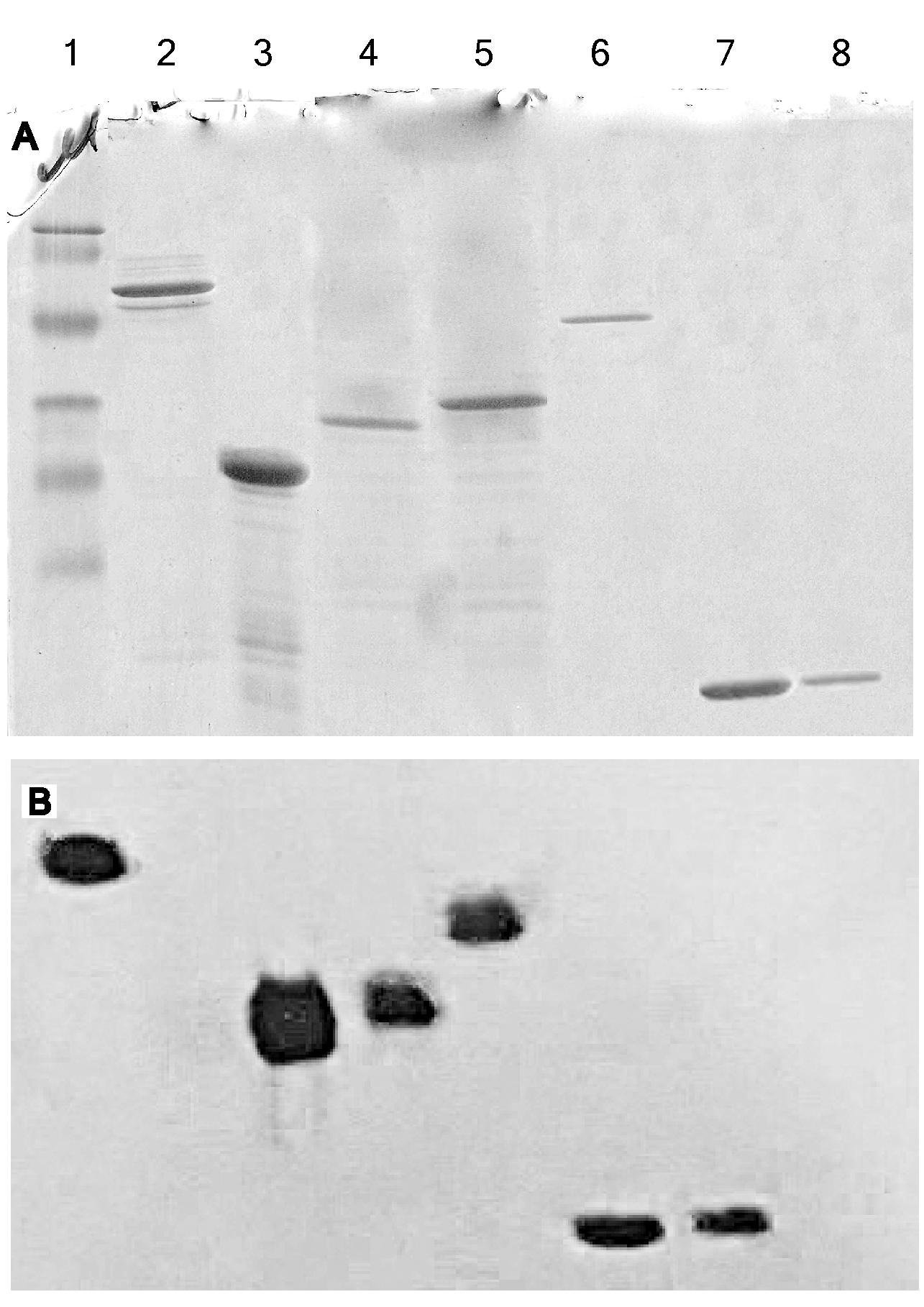


**Supplementary Figure S3.**

**A. SDS-PAGE analysis of purified recombinant proteins.** Lane 1 – Prestained Protein Molecular Weight Market (SM0441, Fermentas), Lane 2 – GST-K17 recombinant protein (73 kDa), Lane 3 – recombinant GST (26 kDa), Lane 4 – GST-S1 recombinant protein (32 kDa), Lane 5 – GST-S4 recombinant protein (34 kDa), Lane 6 – The cleaved product K17 (47 kDa), Lane 7 – The cleaved product S1 (6 kDa), Lane 8 – The cleaved product S4 (8 kDa).

**B. Immunoblotting analysis.** Immunoblot proteins were performed using anti-human keratin 17 monoclonal antibody (MCA1872, Bio-Rad) and rabbit anti-mouse antibody conjugated to horseradish peroxidase (STAR13B, Bio-Rad).


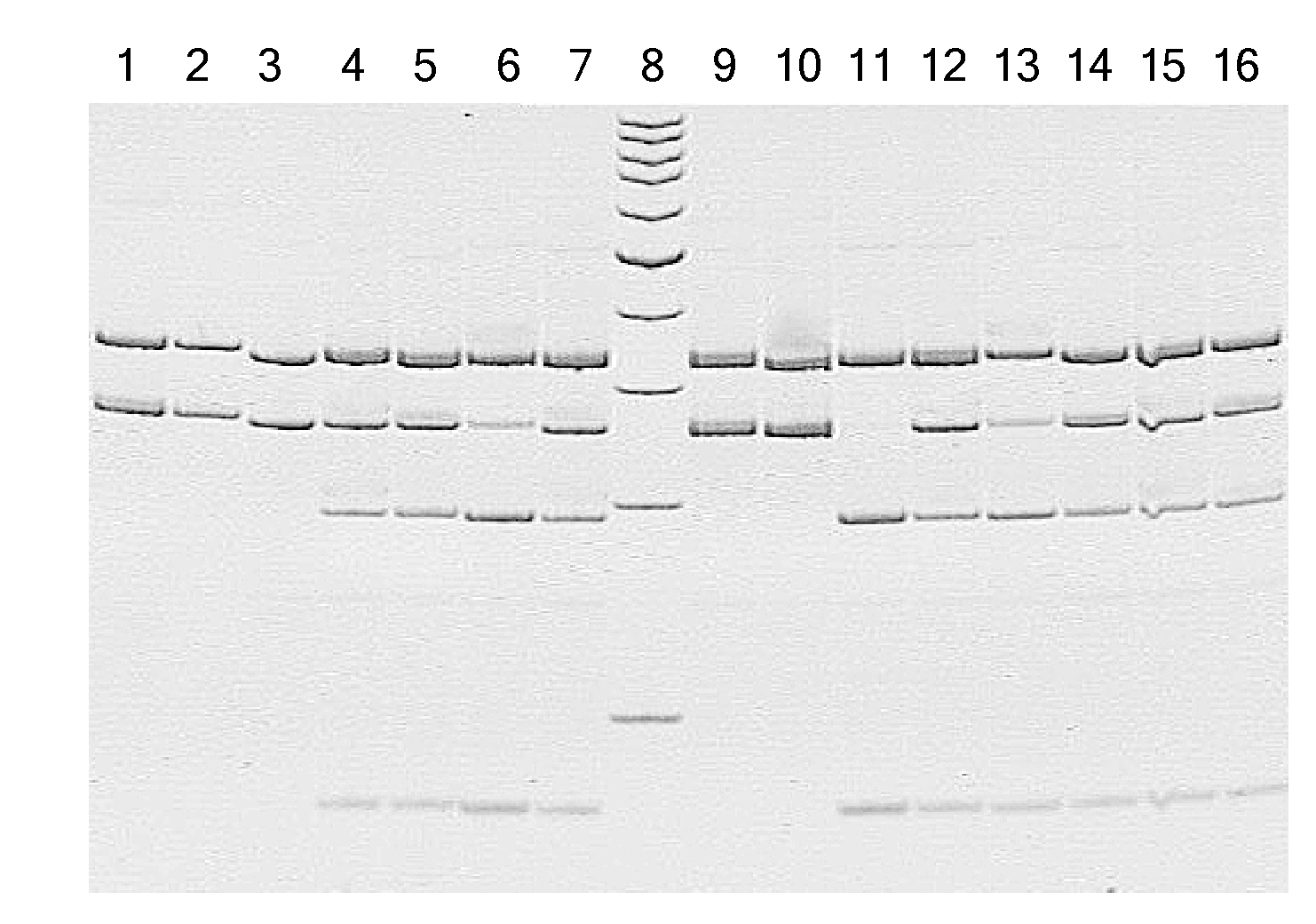


**Supplementary Figure S4. PAGE electrophoresis of *HLA-Cw*06:02*** **PCR products digested by *Sma*I restriction enzyme.** The digested PCR products (*rs1050414*) with *Sma*I generated a 348 bp and 270 bp fragments for allele non-*HLA-Cw*06:02* carriers and 348 bp, 196 bp and 74 bp fragments for allele *HLA-Cw*06:02* carriers. Lane 1 – 3, 9 and 10 genotype non- *HLA-Cw*06:02* carriers, Lane 6, 11, 13 genotype *HLA-Cw*06:02* carriers, Lane 4, 5, 7, 12, and 14-16 heterozygous genotype, Lane 8 – DNA marker (Thermo Scientific GeneRuler 100 bp DNA Ladder, # SM0241).

**Supplementary Figure S5.** Boxplots show T cell proliferation response when treated with K17, the S1 fragment of K17, GST, the S4 fragment of K17, or two synthetic peptides, PS1 and PK. Statistically significant differences between patients and controls are shown in red. The Mann-Whitney test, also known as the Wilcoxon rank sum test, is given as W together with the corresponding p-value.
